# Placement of Dermal Regeneration Template on Fibrotic Dura

**DOI:** 10.7759/cureus.9185

**Published:** 2020-07-14

**Authors:** Garrison A Leach, Hally M Chaffin, Denzil Mathew, Travis Holcombe

**Affiliations:** 1 Surgery, Creighton University School of Medicine, Phoenix, USA; 2 Internal Medicine, Creighton University School of Medicine, Phoenix, USA; 3 Plastic Surgery, Creighton University School of Medicine, Phoenix, USA

**Keywords:** marjolin ulcer, dermal regeneration template, reconstruction, skin graft, craniectomy

## Abstract

We report the case of a male presenting with a large, fungating Marjolin ulcer over a prior craniectomy defect that had undergone several attempts at reconstruction. On presentation, he had a large area of exposed, fibrotic dura that ultimately required excision of the outer layer prior to placement of Integra (Integra LifeSciences, Plainsboro, NJ) and subsequent split-thickness skin grafting. Although there have been four other reports of dermal regeneration templates being used on exposed dura, this is the first case report of one being used on exposed dura that required dural preparation prior to placement. We discuss our rationale for this method of reconstruction, the histology of dermal regeneration template incorporation, and why this approach was necessary to allow for incorporation in this patient.

## Introduction

Full-thickness defects of the scalp pose a unique challenge for the reconstructive surgeon. These cases are made especially challenging when the defect includes the cranium, exposing the underlying dura. There are several options for reconstruction of scalp defects, including local flaps, tissue expansion, and free flaps. Dermal regeneration templates (DRTs), such as Integra (Integra LifeSciences, Plainsboro, NJ), with subsequent split-thickness skin grafts have been used for many years in reconstruction of complicated wounds on various aspects of the body. Integra is an artificial dermis composed of bovine tendon collagen and glycosaminoglycan with an additional silicone epidermal substitute that allows for neodermis ingrowth [1]. 

Although primarily used for reconstruction of burn wounds on the scalp, DRTs have shown to have great utility in coverage of full-thickness defects of numerous etiologies, including oncologic reconstruction. Recently, it has been used with promising results in four prior cases of exposed dura. We present a case of large full-thickness defect with exposed fibrotic dura reconstructed with Integra and subsequent split-thickness skin grafting. This is the first case, to our knowledge, that required surgical preparation of the dura in order to improve Integra incorporation. 

## Case presentation

**Figure 1:**
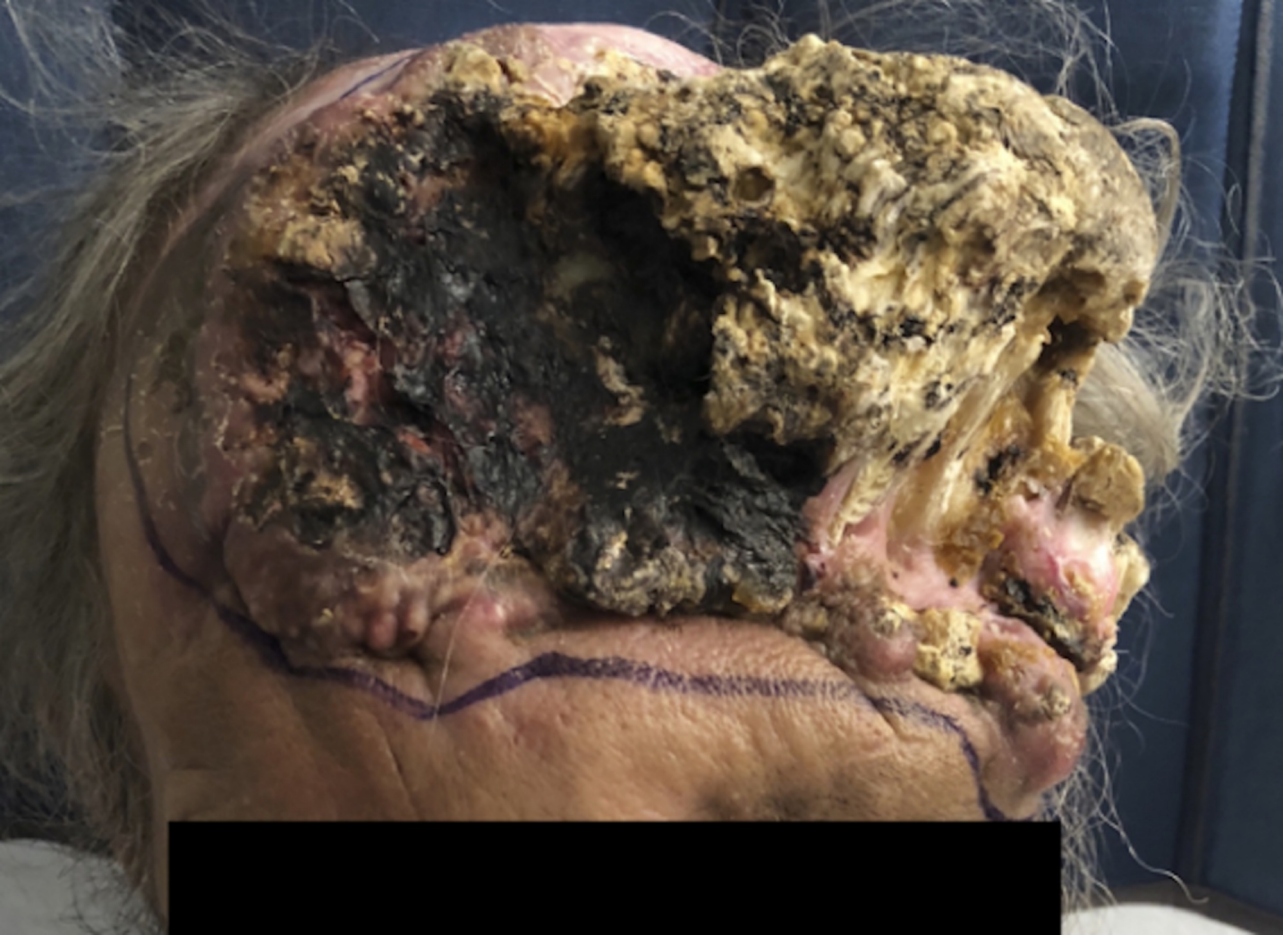
The patient upon presentation.

A 54-year-old-man presented with a fungating scalp mass that had been present for several years (Figure 1). His caregiver noted that it had begun increasing in size over several months prior to presentation. Around 35 years prior, the patient was in a motor vehicle accident that resulted in severe cranial injury requiring craniectomy. He required several surgeries in attempts to reconstruct his scalp and cranium, including alloplastic cranioplasty, several skin grafts, right thigh free flap, and bilateral latissimus free flaps. Ten years prior to presentation, the cranioplasty was removed due to infection. 

**Figure 2:**
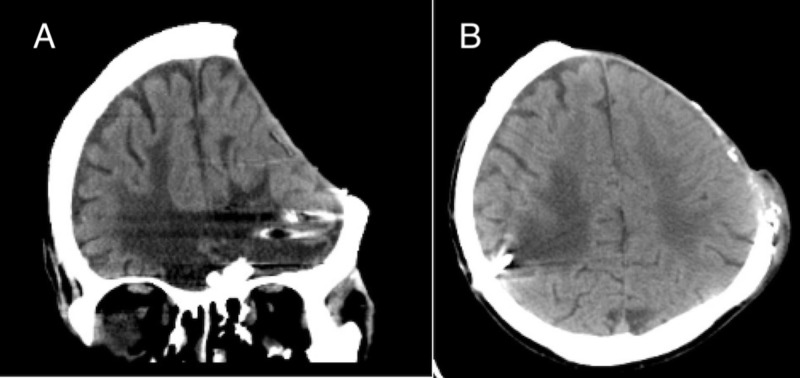
CT images of the patient. In each pane, the patient has radiologic evidence of syndrome of the trephined. However, the presence of this phenomena was difficult to determine clinically as many of his symptoms could also have been secondary to his prior, serious traumatic brain injury. (A) Coronal plane. (B) Axial plane.

On examination, the patient had an oval-shaped 26.5 x 27 cm exophytic calcified scalp mass overlying his prior craniectomy defect. It extended transversely across the frontal region as well as posteriorly in the left temporal and parietal regions. CT and MRI demonstrated no intracranial extension (Figure 2). Biopsy results were consistent with atypical squamous cell carcinoma. A staged excision and reconstruction between neurosurgery and plastic surgery was planned, which included excision with 1.5 cm wide radical margins, placement of Integra synthetic graft, and subsequent skin grafting.

**Figure 3:**
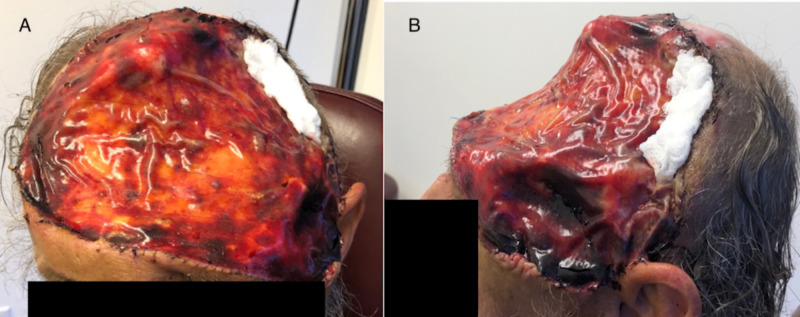
The patient five days after Integra placement. (A) The patient from the anterior perspective. (B) The patient from the left lateral perspective.

During the patient’s first surgery, the lesion was excised primarily off exposed dura but included cranium in the right temporal and parietal regions. Excision off exposed cranium resulted in cranium denuded of periosteum. Permanent pathology confirmed squamous cell carcinoma consistent with a Marjolin's ulcer. Frozen sections, including the dura, confirmed that negative deep margins were obtained. The second surgery began with burring of the outer table of the skull in the areas of exposed cranium. The outer scarred lamella of the dura and granulation tissue was meticulously excised under loupe magnification using an angled curette to expose healthy dural tissue. After inspection, no dural tears were appreciated and Integra was then placed on exposed cranium denuded of periosteum and exposed dura which was 250 cm^2^ in total (Figure 3). The Integra was bolstered with a standard bolster dressing (which was subsequently taken down five days later in clinic). Negative pressure wound therapy was not employed as a bolster in case a dural tear was missed. Of note, during this second surgery the patient was noted to have a separate deformity of the frontal region just superior to his nasal dorsum that was confirmed squamous cell carcinoma. This was not connected to the Marjolin's ulcer of his scalp.

**Figure 4:**
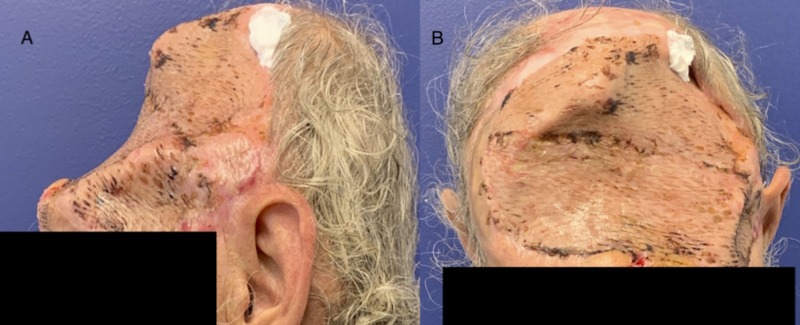
The patient five days post split-thickness skin graft placement. (A) The patient from left lateral perspective. (B) The patient from anterior perspective.

Moreover, pathology noted very different characteristics between the two malignancies. This was ultimately excised and reconstructed with Integra and a split-thickness skin graft as well. The third stage took place four weeks later with removal of the silicone layer of the Integra. The patient had nearly full take of the Integra except at a small area in the left parietal region. Split-thickness skin grafts were harvested from the patient’s thighs, meshed, and inset (Figure 4). 

**Figure 5:**
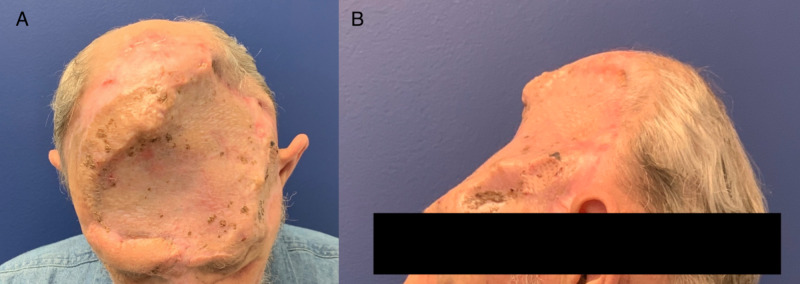
The patient 11 months post split-thickness skin graft/final procedure. (A) The patient from anterior and superior perspective. (B) The patient from left superior and lateral perspective.

At 11 months postop, the patient is doing well and reports no complications. He states that he is satisfied with his outcome thus far (Figure 5). 

## Discussion

The various options available for scalp reconstruction in addition to their indications, benefits, and limitations have been discussed extensively in the literature [2-7]. DRTs were originally employed for scalp reconstruction in full-thickness defects secondary to burn injuries [8]. However, they are also very adroit in the reconstruction of wounds secondary to radiation, trauma, and cancer [9-11]. Moreover, several case reports have discussed the efficacy of Integra in the setting of full-thickness cranial defects when cranioplasty and free flap are not viable options. There have been four case reports attesting to successful incorporation of Integra when placed directly on the dura [11-14]. This case is unique because this patient required surgical preparation of the dura in order to aid Integra incorporation.

In the area with cranium present, our patient had a full-thickness defect with exposed bone denuded of periosteum contraindicating skin grafting. The defect was too large for local flaps, and tissue expansion was not a reasonable choice considering his pathology and desire for quick closure. In order to mitigate donor site morbidity and spare our patient the extensive surgery and recovery he did not desire, free tissue transfer was ruled out for both the area with cranium and the area of prior craniectomy. Additionally, due to his history of failed cranioplasty and his desire to not undergo further major surgery, we decided not to proceed with additional attempts at cranial reconstruction. Therefore, the choice was made to proceed with a two-stage reconstruction with placement of Integra over the exposed cranium and dura with subsequent split-thickness skin graft. The patient did not follow up with an oncologist until after the reconstruction was complete. Thus, plans for adjuvant radiation therapy did not factor into our decision for reconstruction. 

There are four stages to DRT incorporation: imbibition, fibroblast migration, neovascularization, and remodeling and maturation [15]. In our practice, when the cranium is devoid of periosteum, the outer table of the cranium is removed via burring to expose the underlying, highly vascularized diploe in order to aid imbibition and host cell integration into the DRT’s matrix.

To our knowledge, there have been no reports in the literature documenting dural preparation for Integra placement in the setting of extensive dural thickening or scarring. Native dura does not need further wound bed preparation in order to mitigate the risk of damage to the dura. Our patient had extensive dural fibrosis which was most likely due to prolonged exposure of the dura, numerous procedures attempted cranial reconstruction, history of trauma, and infection. Furthermore, on initial excision of the lesion, the patient appeared to have residual methyl methacrylate on his cranium and dura from his prior cranioplasty. Application of methyl methacrylate, a previously common cranioplasty material, can burn and subsequently scar the dura [16]. The primary vascular layer of the dura is a rich plexus on the outer surface [17-19]. In a case of extensive fibrosis, the outer vascular network was most likely compromised. Thus, we decided to meticulously excise the outer lamella of dura. This was to expose any surviving primary anastomotic or penetrating vessels or even to expose a more vascular sublayer such as the secondary anastomotic vessels. Although the anatomy is different, the theory behind this method is comparable to our approach of exposing the diploic space when the patient had exposed denuded cranium. We believe this meticulous dissection and subsequent improved vascularity aided in cellular and vascular ingrowth of the DRT and allowed for successful reconstruction. 

## Conclusions

We present the first known case of Integra placed on exposed dura that necessitated dural dissection. Our outcomes demonstrate the careful excision of the outer layer of a scarred dura can aid with integration of dermal regenerative templates. These results support that Integra is an excellent option for full-thickness scalp defects with exposed dura even in cases of extensive fibrosis.

## References

[1] Clayman MA, Clayman SM, Mozingo DW (2006). The use of collagen-glycosaminoglycan copolymer (Integra) for the repair of hypertrophic scars and keloids. J Burn Care Res.

[2] Desai SC, Sand JP, Sharon JD, Branham G, Nussenbaum B (2015). Scalp reconstruction: an algorithmic approach and systematic review. JAMA Facial Plast Surg.

[3] Leedy JE, Janis JE, Rohrich RJ (2005). Reconstruction of acquired scalp defects: an algorithmic approach. Plast Reconstr Surg.

[4] Hussussian CJ, Reece GP (2002). Microsurgical scalp reconstruction in the patient with cancer. Plast Reconstr Surg.

[5] Mehrara BJ, Disa JJ, Pusic A (2006). Scalp reconstruction. J Surg Oncol.

[6] Ioannides C, Fossion E, McGrouther AD (1999). Reconstruction for large defects of the scalp and cranium. J Craniomaxillofac Surg.

[7] Iblher N, Ziegler MC, Penna V, Eisenhardt SU, Stark GB, Bannasch H (2010). An algorithm for oncologic scalp reconstruction. Plast Reconstr Surg.

[8] Yeong EK, Huang HF, Chen YB, Chen MT (2006). The use of artificial dermis for reconstruction of full thickness scalp burn involving the calvaria. Burns.

[9] Johnson MB, Wong AK (2016). Integra-based reconstruction of large scalp wounds: a case report and systematic review of the literature. Plast Reconstr Surg Glob Open.

[10] Corradino B, Di Lorenzo S, Leto Barone AA, Maresi E, Moschella F (2010). Reconstruction of full thickness scalp defects after tumour excision in elderly patients: our experience with Integra® dermal regeneration template. J Plast Reconstr Aesthet Surg.

[11] Khan MA, Ali SN, Farid M, Pancholi M, Rayatt S, Yap LH (2010). Use of dermal regeneration template (Integra) for reconstruction of full-thickness complex oncologic scalp defects. J Craniofac Surg.

[12] Wain RA, Shah SH, Senarath-Yapa K, Laitung JK (2010). Dermal substitutes do well on dura: comparison of split skin grafting+/− artificial dermis for reconstruction of full-thickness calvarial defects. J Plast Reconstr Aesthet Surg.

[13] de Haas LE, Gardien KL, van Trier AJ, Vloemans AF, Buis DR (2019). The use of Integra in extensive full-thickness scalp burn involving the skull in a child. J Craniofac Surg.

[14] Momoh AO, Lypka MA, Echo A, Rizvi M, Klebuc M, Friedman JD (2009). Reconstruction of full-thickness calvarial defect: a role for artificial dermis. Ann Plast Surg.

[15] Moiemen NS, Vlachou E, Staiano JJ, Thawy Y, Frame JD (2006). Reconstructive surgery with Integra dermal regeneration template: histologic study, clinical evaluation, and current practice. Plast Reconstr Surg.

[16] Drosos GI, Babourda E, Magnissalis EA, Giatromanolaki A, Kazakos K, Verettas DA (2012). Mechanical characterization of bone graft substitute ceramic cements. Injury.

[17] Kashiwagi S, Kato S, Yasuhara S, Wakuta Y, Yamashita T, Ito H (1996). Use of a split dura for revascularization of ischemic hemispheres in moyamoya disease. J Neurosurg.

[18] Protasoni M, Sangiorgi S, Cividini A (2011). The collagenic architecture of human dura mater. J Neurosurg.

[19] Kerber CW, Newton TH (1973). The macro and microvasculature of the dura mater. Neuroradiology.

